# BDNF gene Val66Met polymorphisms as a predictor for clinical presentation in schizophrenia – recent findings

**DOI:** 10.3389/fpsyt.2023.1234220

**Published:** 2023-10-11

**Authors:** Adriana Farcas, Charles Hindmarch, Felicia Iftene

**Affiliations:** ^1^Centre for Neuroscience Studies, Queen’s University, Kingston, ON, Canada; ^2^Providence Care Hospital, Kingston, ON, Canada; ^3^Department of Biomedical and Molecular Sciences, Queen’s University, Kingston, ON, Canada; ^4^Department of Medicine, Queen’s University, Kingston, ON, Canada; ^5^Queen’s Cardiopulmonary Unit, Translational Institute of Medicine, Queen’s University, Kingston, ON, Canada

**Keywords:** schizophrenia, BDNF, gene polymorphism, neurotrophins, genotyping

## Abstract

Schizophrenia is a highly heritable, severe psychiatric disorder that involves dysfunctions in thinking, emotions, and behavior, with a profound impact on a person’s ability to function normally in their daily life. Research efforts continue to focus on elucidating possible genetic underlying mechanisms of the disorder. Although the genetic loci identified to date to be significantly associated with schizophrenia risk do not represent disease-causing factors, each one of them could be seen as a possible incremental contributor. Considering the importance of finding new and more efficient pharmacological approaches to target the complex symptomatology of this disorder, in this scoping review, we are focusing on the most recent findings in studies aiming to elucidate the contribution of one of the genetic factors involved – the BDNF gene Val66Met polymorphisms. Here we performed a systematic search in Pubmed, Embase, and Web of Science databases with the search terms: (BDNF gene polymorphism) AND (schizophrenia) for articles published in the last 5 years. To be selected for this review, articles had to report on studies where genotyping for the BDNF Val66Met polymorphism was performed in participants diagnosed with schizophrenia (or schizophrenia spectrum disorders or first-episode psychosis). The search provided 35 results from Pubmed, 134 results from Embase, and 118 results from the Web of Science database. Twenty-two articles were selected to be included in this review, all reporting on studies where an implication of the BDNF Val66Met polymorphisms in the disorder’s pathophysiology was sought to be elucidated. These studies looked at BDNF gene Val66Met polymorphism variants, their interactions with other genes of interest, and different facets of the illness. The Met/Met genotype was found to be associated with higher PANSS positive scores. Furthermore, Met/Met homozygous individuals appear to present with worse cognitive function and lower levels of serum BDNF. In the Val/Val genotype carriers, increased BDNF levels were found to correlate with weight gain under Risperidone treatment. However, due to heterogeneous results, the diversity in study populations and studies’ small sample sizes, generalizations cannot be made. Our findings emphasize the need for further research dedicated to clarifying the role of gene polymorphisms in antipsychotic treatment to enhance specificity and efficacy in the treatment of schizophrenia.

## Introduction

Schizophrenia is a severe, chronic mental illness involving thinking, emotions, and behavior dysfunctions, resulting in impaired perception, social withdrawal, and loss of touch with reality. Individuals with schizophrenia often experience hallucinations, delusions, disorganized speech, and abnormal movements or behaviors. Pharmacological treatments available for schizophrenia vary in efficacy, with an estimated 34% of patients meeting the criteria for treatment resistance (1). Considering the profound impact this condition has on a person’s life, requiring long-term treatment and care, research efforts continue to focus on elucidating possible underlying mechanisms of the disorder. Biological, genetic, psychological, and environmental factors have been studied over the years. In the realm of genetic research advances, the genetic loci identified to be significantly associated with schizophrenia risk do not necessarily imply a causality link, but they can be each seen as a possible contributor factor (2). In line with ongoing efforts at exploring pharmacological targets with direct clinical applicability, in this scoping review, we focus on the most recent findings in studies aiming to elucidate the contribution of one of the genetic factors – the BDNF gene Val66Met polymorphisms.

The brain-derived neurotrophic factor (BDNF) is one of the most studied members of the neurotrophin family, which includes neural growth factor (NGF) and neurotrophins 3 and 4, among others (3). BDNF gene expression is highest in the brain (in the hippocampus, amygdala, cerebral cortex, and hypothalamus) as reviewed by Pruunsild (4), but mRNA encoding BDNF is also found in peripheral tissues including the heart, lung, prostate, and bladder (5). The BDNF gene contains 11 exons and is complemented by antiBDNF expression, which has been shown to form dsRNA duplexes in the brain, implying layered regulation of this important factor. BDNF is a secretory polypeptide released at the synapse, affecting synaptic plasticity (6). Binding to a tropomyosin receptor kinase B, also known as tyrosine kinase receptor B (TrkB), BDNF becomes an important signaling factor implicated in regulating proliferation, differentiation, survival, and death of neuronal and glial cells, as well as long-term memory formation, the regulation of long-term potentiation, and hippocampal synaptic plasticity (7, 8). The dysregulation of the TrkB/BDNF pathway has also been implicated in neurological and neurodegenerative conditions as well as stress-related disorders (9–11).

Alterations in BDNF at both the gene and protein levels during brain development have been implicated in the myriad of abnormalities found in the brains of individuals with schizophrenia (12–14). The genome-wide association study of schizophrenia (GWAS) confirmed the relevance of BDNF, identifying the BDNF genomic locus as enriched in common single nucleotide polymorphisms (SNPs) associated with the disorder, although not reaching the genome-wide statistical threshold (2). However, from the perspective of the neurodevelopmental hypothesis of schizophrenia, which considers the utmost complexity of numerous factors and their interactions in the etiology of this disorder, BDNF has been proposed as a candidate to explain part of the pathogenesis of this disease (13). Located on chromosome 11 in humans, the BDNF gene has a complex structure, encompassing 11 different exons and nine promoters (4). A non-synonymous polymorphism in this gene - rs6265 (C → T, Val → Met), also called Val66Met or G196A polymorphism, is common (15). An amino acid substitution - valine (Val) to methionine (Met) at codon 66 leads to profound impairment in the neuronal activity-dependent secretion of BDNF (16) as the BDNF gene is one of the few genes that are prominently implicated in cognitive functions (14). The exact mechanisms that lead to BDNF alterations in schizophrenia are still largely unknown. However, numerous studies hint at epigenetic changes at the BDNF gene locus, providing a link between genes and environment, particularly stress, childhood adversities, and inflammation (17). The Met allele has been historically considered to confer the disadvantaged phenotypes – at the cellular, structural, physiological, and behavioral levels (6). Episodic memory has been shown to be impaired in healthy individuals carrying the Met allele (18) and substantially impaired in Met/Met carriers – among individuals with schizophrenia, siblings, and healthy controls, compared to other genotypes (16, 19). In schizophrenia, it has been suggested that BDNF signaling may critically affect the structure and functioning of neural circuits involved in modulating neurotransmitter systems, including dopaminergic, serotoninergic, and GABAergic (14, 20). In other studies, individuals carrying the Val/Val allele, initially thought as protective, were also identified as at risk for developing schizophrenia and, in patients, being at risk of experiencing more severe symptoms than patients carrying at least one Met allele (21, 22). An interplay between one allele or the other and environmental factors may explain these mixed, conflicting findings and justify efforts to clarify the current understanding of genetic polymorphism’s impact on schizophrenia.

In this review, we aim to capture the current consensus and divergence in findings relating to the implication of the BDNF Val66Met polymorphisms in the clinical presentation of individuals who have schizophrenia. Data supporting the hypothesis that peripheric BDNF gene expression and protein levels reflect those in the brain hints at possible avenues of finding applicability of genetic polymorphism determination for prognosis, therapy monitoring and therapeutic response in schizophrenia (23).

## Methods

**Figure 1:**
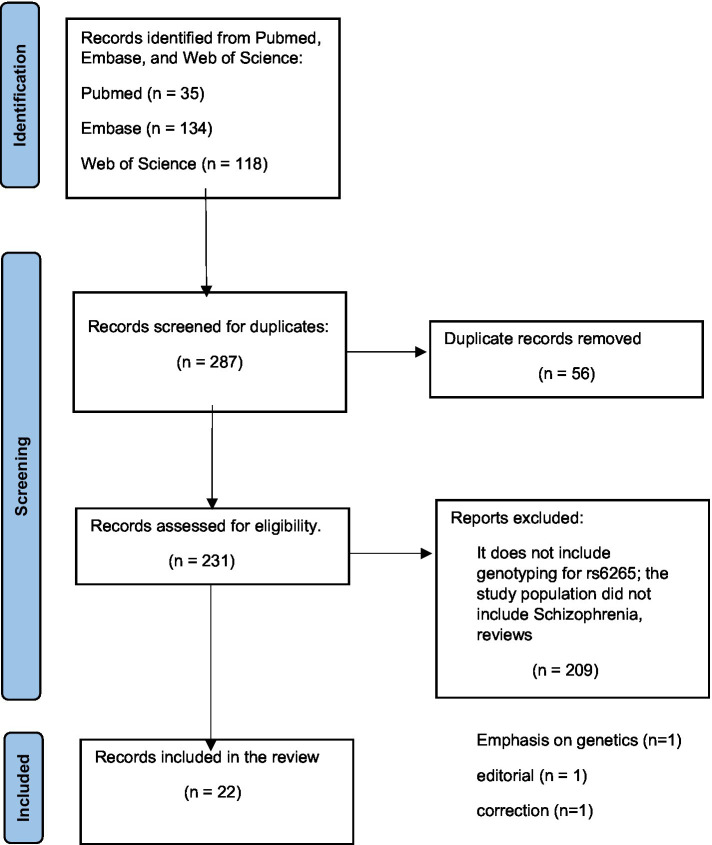
PRISMA diagram.

Our team systematically searched Pubmed, Embase, and Web of Science databases with the search terms: (BDNF gene polymorphism) AND (schizophrenia) for articles published in the last 5 years. The search provided 35 results from Pubmed, 134 results from Embase, and 118 results from the Web of Science database. To be selected for this review, articles had to report on studies where genotyping for the BDNF Val66Met polymorphism was performed in participants diagnosed with schizophrenia or schizophrenia spectrum disorders or first-episode psychosis. In addition, these studies also had to aim at analyzing for associations between the Val66Met polymorphism and risk for schizophrenia or with the clinical presentation of participants. After reading abstracts and removing articles that did not fit the criteria, 22 articles were selected to be included in this review seen in the PRISMA diagram (Figure 1).

## Results

Twenty-two articles were selected for this scoping review, all reporting on studies involving participants with a schizophrenia or schizophrenia-related disorder, where an implication of the BDNF Val66Met polymorphisms in the disorder’s pathophysiology was sought to be elucidated. These studies looked not only at different polymorphism variants but also their interactions with other genes of interest and at different facets of the illness, in some instances highlighting associations with its most difficult symptoms – the negative ones. Table 1 summarizes the 22 studies with their population, aim, outcome measures, and main findings.

**Table 1:** Description of studies included in this review, with main outcome measures and findings.

Study	Study population and aim	Outcome measures	Main findings
1. Binbay et al. (24)	*n* = 52 individuals with schizophrenia (SZ) and other psychotic disorders *n* = 104 individuals with psychotic experiences *n* = 87 individuals with subthreshold psychotic experiences *n* = 194 individuals with no history of psychotic experiences Participants from Turkey Aim: to evaluate the association between COMT/BDNF polymorphisms and the extended psychosis phenotype	BDNF rs6265 was genotyped in every participant; COMT rs4680 was genotyped in 366 individuals (due to financial limits).	There was no significant association between BDNF Val66Met and COMT Val158Met polymorphisms and the extended psychosis phenotype; The frequency of Met carriers in the BDNF rs6265 genotype was slightly higher in individuals with subclinical psychotic experiences than in the group with no psychotic experiences.
2. Kim and Kim (25)	*n* = 157 patients with schizophrenia *n* = 241 healthy controls Participants from the Republic of Korea Aim: to explore the contribution of BDNF polymorphisms to susceptibility to SZ; to explore associations between specific BDNF gene alleles and genotypes with specific clinical presentations.	Genotyping performed for 2 BDNF SNPs: 196G/A(rs6265) and 11757G/C(rs16917204) PANSS BPRS K-CDSS	For genotype and allele distribution, no significant association with SZ was found; SNP genotypes could not predict clinical severity in schizophrenia patients; The existence of A allele in 196G/A correlated with a personal history of suicide attempts.
3. Schweiger et al. (26)	*n* = 85 healthy volunteers in Discovery sample *n* = 253 healthy volunteers in Replication sample *n* = 58 unaffected first-degree relatives of patients with SZ in Relatives sample Participants from Germany Aim: to examine the influence of the functional BDNF SNP rs6265 (Val66Met) on cognitive control; to clarify underlying mechanisms using fMRI	Genotyping for BDNF SNP rs6265 (Val66Met) fMRI	Significant increase in interregional connectivity between ACC and PFC in the BDNF Met allele carriers. Significant increase in the connectivity between ACC and the medial parts of PFC BA9 in the healthy first-degree relatives of patients with SZ.
4. Xia et al. (27)	*n* = 825 patients with chronic SZ, of which *n* = 123 with and *n* = 702 without suicide attempts *n* = 445 healthy controls without a history of suicide attempts Participants from the Chinese Han population Aim: to explore the association between suicidality and the functional polymorphism of BDNF Val66Met in patients with SZ	Genotyping of BDNF Val66Met polymorphisms PANSS	BDNF Val66Met was not found to contribute directly to susceptibility to SZ. The BDNF Val66Met polymorphism appears to be associated with a history of suicide attempts in SZ; the Val66 (G196) allele and corresponding Val/Val genotype were significantly more frequent in patients with SZ with lifetime suicide attempts than in patients without lifetime suicide attempts. BDNF genotype and age and cigarettes smoked daily were associated with suicide attempts in SZ.
5. Zhang et al. (28)	*n* = 694 patients with schizophrenia *n* = 725 healthy controls Aim: to characterize the association of BDNF rs6265 and TNF-α rs1799964 polymorphisms with SZ in Han Chinese individuals.	The SNPs rs6265 and rs1799964 were genotyped PANSS RBANS	No significant association of the BDNF rs6265 polymorphism with SZ; The interaction of the BDNF and TNF-α genes contributes to cognitive dysfunction in SZ, possibly by decreasing the expression of BDNF in the hippocampus.
6. Kirli et al. (29)	*n* = 366 individuals with psychotic experiences (PE) and psychotic disorders (PD) *n* = 254 re-evaluated at follow-up 6 years later All participants from Turkey Aim: to investigate longitudinal associations between BDNF Val66Met polymorphism and different levels of the extended psychosis phenotype	BDNF Val66Met polymorphism genotyped	The BDNF Val/Val genotype was found to be associated with the extended psychosis phenotype.
7. Sosin et al. (30)	*n* = 130 patients with treatment-resistant schizophrenia Participants were divided into TRS (treatment-resistant) and nTRS (non-treatment-resistant) Participants from Saint Petersburg, Russia Aim: to identify the roles of DRD2, DRD3, HTR2A, BDNF, and CYP2D6 genetic polymorphisms in the presence of cognitive impairment in patients with SZ, depending on the presence or absence of treatment resistance.	Genotyping for DRD2, DRD3 HTR2A, BDNF and CYP2D6 genetic polymorphisms PANSS GAF BACS	No association between the studied gene polymorphic variants and TRS was found. The polymorphic variant CYP2D6*4 showed an effect on cognitive function in the TRS group.
8. Veras et al. (31)	*n* = 56 patients with schizophrenia or schizoaffective disorder *n* = 20 healthy controls Participants from Brazil Aim: to explore the relationship between early trauma and cognition in a sample that included BDNF Val66Met and Val66Val polymorphism	Genotyping for BDNF Val66Met polymorphism PANSS WAIS-III ETI HAM-D HAM-A Young Mania Rating Scale	A significantly greater relationship was found between physical abuse and perceptual organization among Met allele carriers than Val allele carriers. A strong negative correlation was found between IQ scores and early trauma scores in the Met allele carriers only. Perceptual Organization Index (WAIS) negatively correlated with general trauma in the Met allele carriers only. No significant correlations were found between WAIS indices and traumatic experiences within the Val66Val subgroup.
9. Fu et al. (32)	*n* = 1,407 patients with schizophrenia *N* = 1,136 healthy controls Participants from the Han Chinese population Aim: to elucidate the role of the BDNF gene (rs6265)	Genotyping for BDNF rs6265 polymorphism	A positive association was found between rs6265 and SZ, with the A allele of rs6265 conferring a protective effect.
10. Pujol et al. (33)	*n* = 98 individuals with non-affective first episode psychosis (FEP) *n* = 117 healthy controls Participants from Spain Aim: to explore the individual and interactional effects of the Val66Met and C677T polymorphisms on hippocampal atrophy in FEP	Genotyping of BDNF gene Val66Met and MTHFR geneC677T polymorphisms Magnetic resonance imaging for hippocampal volume	A bilateral hippocampal shrinkage was found in patients with FEP. An Interactional effect was found between the BBDNF and MTHFR gene polymorphisms on hippocampal volume in patients with FEP and HC. BDNF Met carriers with the CT-TT genotype showed decreased hippocampal volume compared to individuals with the homozygous CC genotype.
11. Mitra et al. (34)	*n* = 50 patients with schizophrenia n = 50 healthy controls Participants from India Aim: to determine the effect of BDNF rs6265 polymorphism on serum BDNF levels in SZ	Serum BDNF levels Genotyping for BDNF rs6265 polymorphism PANSS GAF	Serum BDNF levels were decreased significantly in patients with SZ compared to healthy controls. Serum BDNF levels were significantly correlated with the age, age of onset of SZ and GAF scores. No significant difference between the 3 genotypes and the serum BDNF levels. No significant association between the clinical scores (PANSS and GAF) with the BDNF rs6265 polymorphism.
12. Suchanek-Raif et al. (35)	*n* = 401 patients with schizophrenia *n* = 657 healthy controls Participants of Caucasian Polish origin Aim: to evaluate the potential association between five SNPs of the TrkB gene and SZ and to estimate any interaction with SNPs of the rs6265 BDNF gene.	Genotyping for TrkB gene SNPs: rs1867283, rs10868235, rs1565445, rs1387923, rs2769605 BDNF rs6265 polymorphism PANSS	The rs1387923 and rs10868235 SNPs are associated with a risk of SZ. The G/G polymorphic genotype of rs1387923 and the T/T polymorphic genotype of rs10868235 had a higher risk of SZ in men compared to women. In men, the A/A wild genotype of rs1387923 was found to be protective compared with the G/G polymorphic genotype. Sex differences in the risk of developing SZ for the polymorphisms of the TrkB and BDNF genes. The ATAAT haplotype was associated with a lower risk of SZ for men; the GTAGCG haplotype (built with the 5 SNPS in TrkB gene and rs6265 BDNF gene) was associated with a lower risk of SZ for women.
13. Xu et al. (36)	*n* = 573 patients with schizophrenia treated with antipsychotics From Beijing, China Aim: Explore the influence of BDNF and the Val66Met (rs6265) polymorphism on the association of age of onset, cognitive function, and clinical symptoms in schizophrenia	Serum BDNF level Val66Met Polymorphism genotyping Symptoms assessed with PANSS Cognitive measure: RBANS	Worse cognitive function was associated with a high level of negative symptoms and a low level of serum BDNF. A significant interaction between the age of onset and BDNF level. Negative symptoms were found as a full mediator between the age of onset and cognitive function in Met homozygous patients. Negative symptoms partially mediate the relationship between age of onset and cognitive function in Val/Met heterozygous patients. No such mediator effect in Val homozygous patients.
14. Abbasian et al. (37)	*n* = 71 unrelated patients with schizophrenia *n* = 88 healthy controls All participants from Iran Aim: to determine whether BDNF rs6265 is associated with SZ, its psychopathology and IQ.	rs6265 genotyped PANSS WAIS	BDNF polymorphism significantly increased the risk of SZ in different genotypes; Significant increase in C allele (Val) frequency in patients with SZ compared with controls; Significant increase in CC (Val/Val) genotype in SZ patients
15. Karacetin et al. (38)	*n* = 101 individuals with early-onset schizophrenia (EOS) or other psychotic spectrum disorders (SSD) *n* = 150 healthy controls Participants from Turkey Aim: to investigate clinical characteristics of adolescents with EOS in relation to BDNF Val66Met (rs6265) or DRD2/ANKK1 Tag1A (rs1800497) polymorphisms	Genotyping for BDNF Val66Met (rs6265) and DRD2/ANKK1 Tag1A (rs1800497) polymorphisms PANSS	EOS/SSD group with Val66Met heterozygote allele revealed lower levels of negative symptoms than the Val/Val homozygote. SSD patients who carried the Lys/Glu polymorphism had higher levels of substance use than Glu/Glu carriers (DRD@/ANKK1 TAG1A).
16. Liu et al. (39)	*n* = 225 antipsychotic drug naïve first episode (ANFE) patients *n* = 125 unrelated healthy controls All patients and controls were Han Chinese 12-week longitudinal trial – patients treated with Risperidone to evaluate the relationship between weight gain and BDNF level	BDNF Val66Met polymorphism was genotyped PANSS Weight change	No significant association between Val66Met genotype and weight gain induced by risperidone treatment after controlling for age, baseline BMI and reduction in PANSS total score; After treatment, patients showed a non-significant increase in BDNF levels; Increased BDNF levels were correlated with weight gain in Val/Val homozygote patients; A significant association between the baseline BDNF levels and weight gain induced by risperidone treatment in patients with Val/Val genotype.
17. Morozova et al. (40)	*n* = 655 patients with schizophrenia, Persistent delusional disorders, acute and transient psychotic disorders and schizoaffective disorders *n* = 768 healthy controls All participants from Moscow, Russia Case–control study aiming to research the associations between symptomatology and SNP of genes related to neurotransmission and neurotrophic factor systems in patients with SZ.	SNPs of BDNF rs6265, DRD# rs6280, HTR1A rs6215 and 5HT2A rs7322347 genotyped; Immunological parameters assessed in serum; PANSS FAB BFCRS	No significant associations of SNPs relationship with PANSS; Patients with genotype T/T (Met66Met polymorphism) demonstrated higher scores, reflecting more severe symptoms, regardless of age; Patients with genotype T/T (Met66Met) showed lower FAB scores; No statistically significant associations between genotype and immune status parameters were found.
18. Su et al. (41)	*n* = 262 drug-naïve first episode patients (DNFE) with schizophrenia; *n* = 844 patients with chronic schizophrenia; *n* = 1,043 healthy control; From Beijing, China Aim: to investigate the effect of BDNF polymorphisms on cognitive deficits in DNFE and chronic patients with SZ	4 SNPs in BDNF gene were genotyped (rs6265, rs12273539, rs18035210, rs2030324) Symptoms assessed with PANSS Cognitive measure: RBANS	No association between BDNF SNPs and SZ; Positive association (weak effect) between BDNF polymorphism and cognition (rs12273539); Cognitive function was lower in DNFE patients with rs2030324 TT and TC genotype than HC; Cognitive function is lower in chronic patients with rs2030324 TT and TC genotype than DNFE;
19. Kaya et al. (42)	*n* = 102 patients with schizophrenia *n* = 98 healthy controls Participants from Turkey Aim: to investigate the relationship between the BDNF gene Val/Met polymorphism and clinical symptoms, attention and executive functions in patients with SZ	Genotyping of BDNF Val66Met polymorphisms PANSS WCST Stroop test	No difference in genotype and allele distributions between the patients and the control group. No relationship was found between the age of onset and the genotypes of the BDNFVal66Met polymorphism. No significant difference was found between Val/Val and Met allele genotypes in WCST subscale scores in patients with SZ. Poor performance in the Stroop test was determined in those with the Met allele compared to Val allele in the patient group.
20. Pan et al. (43)	*n* = 55 patients with schizophrenia *n* = 50 healthy controls Participants from the Han Chinese population Aim: to evaluate the relationship between the BDNF and MMP-9 SNPs and their correlation with clinical features of SZ	Genotyping of the BDNF and MMP-9 SNPs PANSS	Patients were carrying a significantly higher frequency of the BDNF rs6265 SNP GG/GA genotypes and a lower frequency of the AA genotype compared to healthy controls. BDNF GG genotype showed significantly higher PANSS and positive symptoms scores than GA and AA genotypes; AA genotypes showed significantly lower scores than GA genotype. MMP-9 CC genotype showed significantly higher PANSS and general scores than CT and TT genotypes but not in positive or negative scores.
21. Pilla et al. (44)	*n* = 125 patients with schizophrenia *n* = 50 healthy controls Participants from India Aim: to determine the frequency of BDNF rs6265 polymorphism and its association with SZ	Genotyping of BDNF rs6265 polymorphism PANSS GAF	Significant association of BDNF genetic polymorphism Val66Met with SZ. The A allele of BDNF val66met gene is associated with increased risk for SZ.
22. Ping et al. (45)	*n* = 134 patients with schizophrenia *n* = 64 healthy controls Participants from Zhongshan City, China Aim: to investigate the association between BDNF and CREB gene polymorphisms in SZ	Genotyping for BDNF gene SNPs rs11030101, rs2030324, rs6265, and the CREB gene SNPs rs6740584, rs2551640 PANSS	Compared with controls, there was a significant increase in the rs11030101, rs2030324, and rs6265 AAC haplotype frequency in patients with SZ. Genotypes at the rs11030101 and rs6265 loci of BDNF gene were associated with either negative or clinical pathological symptoms.

BACS, Brief Assessment of Cognition in Schizophrenia; BFCRS, the Bush –Francis Catatonia Rating Scale; CREB, Cyclic adenosine monophosphate response element binding protein; ETI, Early Trauma Inventory; FAB, Frontal Assessment Battery; GAF, Global Assessment of Functioning; HAM-A, Hamilton A; HAM-D, Hamilton Depression; K-CDSS, Korean version of the Calgary Depression Scale for Schizophrenia; PANSS, the Positive and Negative Syndrome Scale; RBANS, Repeatable Battery for the Assessment of Neuropsychological Status; SZ, Schizophrenia; WAIS, Wechsler Adult Intelligence Scale; WCST, the Wisconsin Card Sorting.

## Discussion

Since the first study announcing the isolation of BDNF as a new neurotrophic factor in the pig brain in 1982 (46), a consistently increasing number of researchers have continued to explore its role in the physiology and pathology of the human brain. Although studies selected for this review were highly prioritized based on narrow selection criteria that included only the most recent publications (within the past 5-years), they reflect findings from different geographical regions and ethnicities, painting a complex interplay of factors in addition to the BDNF gene, that contribute to the pathophysiology of schizophrenia. Considering that ethnicity has been cited as an important factor explaining divergent findings of the associations between the BDNF Val66Met polymorphism and psychiatric disorders (47, 48), it is worth mentioning that the study populations included here belong to different ethnic backgrounds: eight of the studies were performed in China, 2 in India, 4 in Turkey, 2 in Russia, and 1 in Spain, Brazil, Germany, Iran, South Korea, and Poland, respectively. Significant ethnic differences in the BDNF Val66Met polymorphism were suggested to arise from a natural selection of a particular allele and many environmental factors, further complicating the inferential analysis (49).

Recent findings also suggest that gender and age impact how the BDNF Val66Met polymorphism contributes to neurodegenerative disease (50). The study populations included here consist of adults - 18 to 65 years old, except for Karacetin (38). where participants were adolescents with early-onset schizophrenia or other psychotic spectrum disorders. The primary diagnosis of participants encompasses schizophrenia and schizophrenia spectrum disorders, as well as other psychotic spectrum disorders as a subcategory for comparison – as in Karacetin (38). Kirli (29) studied individuals with psychotic experiences and psychotic disorders, including schizophrenia, to investigate longitudinal associations between the BDNF Val66Met polymorphism and different levels of the extended phenotype. Another study compared genotyping differences between healthy volunteers and unaffected first-degree relatives of patients with schizophrenia (26).

Samples varied amply in size, from *n* = 50 in kumar (34) to *n* = 1,407 in Fu (32). In most of the studies, the gender variable was predominantly represented by males, with a few exceptions where both genders were matched or females slightly higher in numbers (24, 26, 34). Considering the established understanding that differences in the epidemiology of psychiatric disorders and the persisting tendency to underrepresent women as participants in studies (51), it is worth highlighting that equal recruitment of genders would ensure greater generalizability of findings (52). Although sex as a distinguishing biological factor is crucial in genetic studies, the social construct of gender may further bring clarity, especially in the light of research highlighting more prevalent gender dysphoria in individuals with schizophrenia than in the general population (53). Sex differences were found in the risk of developing schizophrenia by Shuchanek-Raif (35). In this study, the ATAAT haplotype (built with 5 SNPs in the TrKB gene) was associated with a lower risk for schizophrenia in men. In comparison, the GTAGCG haplotype (built with the 5 SNPs in the TrkB gene and rs6265 BDNF gene) was associated with a lower risk of schizophrenia for women.

Most studies included a healthy control group, except those looking for associations between the BDNF Val66Met polymorphism and clinical facets of schizophrenia or the extended psychosis phenotype (29, 36), or evaluating genetic implications in treatment resistance (30).

The clinical severity of the illness extended from the drug-naïve first episode of psychosis to treatment-resistant schizophrenia symptoms and a history of suicide. Clinical presentation is consistently assessed and highlighted in most of the studies. A significant association between the BDNF Val66Met polymorphism and symptoms was found in Pan (43), where the BDNF GG (Met/Met) genotype showed higher Positive and Negative Syndrome Scale (PANSS) positive symptoms score compared to the GA (Met/Val) and AA (Val/Val) genotypes. The AA genotypes showed significantly lower scores than the GA genotype in this study. Negative symptoms were also found to be a full mediator between the age of onset and cognitive function in Met homozygous patients and a partial mediator in Val/Met heterozygous patients by Xu (36). This same study (36) connects worse cognitive function with increased negative symptoms and a low level of serum BDNF. Systematic reviews focusing on peripheral levels of BDNF (54, 55) concluded that moderate reductions of BDNF are observed both in drug-naïve and medicated individuals. Certain BDNF isoforms at low levels were consistently found to correlate with cognitive impairment in individuals with schizophrenia compared to controls (56). In line with these findings, Morozova (40) found that patients with genotype T/T (Met66Met polymorphism) demonstrated higher scores on PANSS, reflecting more severe symptoms, regardless of age. None of the other studies found a significant association between positive and negative PANSS scores and the Val66Met polymorphism, most focusing on the risk for schizophrenia or association with cognitive functions. The PANSS was one of the most utilized clinical assessment tools, followed by BACS, and BFCRS, among others. One of the studies also included the HAM-A and HAM-D to address the affective component of the schizoaffective disorders portion of its sample (31).

Suicidality, a critical issue in schizophrenia, and its association with the BDNF Val66Met polymorphism were explicitly investigated by Xia (27). Their findings show a clear association between this genetic polymorphism and a history of suicide attempts in schizophrenia. Interestingly, the val66 allele and the corresponding Val/Val genotype were more frequent in patients with schizophrenia presenting with lifetime suicide attempts. This study also found an association between the BDNF genotype, age, cigarettes smoked per day, and suicide attempts in patients, highlighting the interaction between genetic and environmental factors in suicidal behavior among patients with schizophrenia.

Cognition was the focus of explorations in five of the studies, depicting poor cognitive performance in patients with the Met allele compared to the Val, in Kaya (42).

A negative correlation between IQ scores and early trauma in the Met allele carriers, as well as a negative correlation between perceptual organization and trauma in the same group, was found by Veras (31). Worse cognitive function was associated, as expected, with a high level of negative symptoms and a low level of serum BDNF in Xu (36), with a mediator effect of the negative symptoms for the interaction age of onset – cognition in Met homozygous patients, as mentioned previously. Looking at the association between the BDNF rs6265 and the TNF-α rs1799964 polymorphism in patients with schizophrenia, Zhang (28) found that the interaction of these two genes contributes to cognitive dysfunction, possibly by decreasing the expression of BDNF in the hippocampus. Only one study – Morozova (40) assessed immunological parameters along with their primary assessments, finding no association between genotype and immune status of patients, contrary to recent literature suggesting the contrary (3). Patients with schizophrenia have been found in numerous studies to present with upregulated genes for inflammatory cytokines and downregulated BDNF gene transcription (57, 58).

Notably, two studies involved fMRI in their design, aiming to delineate morphological and functional changes related to the BDNF Val66Met polymorphism. Schweiger (26) found a significant increase in interregional connectivity between ACC and PFC in the BDNF Met allele carriers. In contrast, Pujol (33) found that BDNF Met carriers with the MTHFR gene CT-TT genotype presented with decreased hippocampal volume.

Multiple genetic interactions were explored by Su (41), Morozova (40), Zzhang (28), Kim and Kim (25), Sosin (30), Pan (43), Karacetin (38), Ping (45), and Suchanek-Raif (35), revealing various associations or propensity for risk for schizophrenia.

An interesting and pragmatic approach was taken by Liu (39), where the study focused on evaluating the relationship between BDNF levels and the BDNF genotype and weight gain in individuals with schizophrenia treated with Risperidone. While a non-significant increase in BDNF levels correlated with weight gain in Val/Val homozygous patients, these findings raise the importance of examining genetic factors underlying therapeutic response.

A recent review of evidence demonstrating that dysregulation of neurotrophic signaling is common to most neurological and neurodegenerative disorders highlights the importance of restoring the BDNF/TrkB pathway by its fine-tuned activation or suppression (11). The authors discuss and evaluate different strategies directed to increase the availability of BDNF, from systemic BDNF administration and its limitations, nanoparticle-mediated transport, gene therapy with BDNF-encoding viral vectors or transplantation of BDNF-releasing cells, to antidepressant medication and exercise, stressing that in the end the efficiency of such treatments could be limited when receptor stability and function are aberrant, as it is the case in psychiatric disorders.

Considering the wide range of findings in the studies selected for this review, *the limitations* in generalization, besides study design and methodology variations, are imposed by the complexity of possible factors affecting the BDNF Val66Met polymorphism. Interethnic genetic differences remain significant, and specifying the country of origin may not cover ethnicity. Studies show, for example, that the Met allele frequency is around 50% in the Chinese population but only around 20% in Caucasian subjects (27, 59). In the current globalization era, samples from any geographical region may include different ethnicities. Serum BDNF level was also found to have a diurnal as well as seasonal variation (60), and a diurnal variation in women appears to be correlated with the ovarian function (61).

*Further directions* may regard longitudinal studies, samples that are representative of the number of participants, sex, and gender (including those who have undergone gender-affirming treatment), as well as ethnicity, in-depth exploration of associations between the BDNF Val66Met polymorphism, its TrkB receptor and immune factors as well as peripheral levels of BDNF, and all this taking into account morphologic changes depicted through imaging investigations as well as the diurnal, seasonal and hormonal BDNF variations, medication interactions, metabolic and lifestyle factors. Identifying BDNF gene variants that lead to effective antipsychotic drug response could have crucial implications for patients and the clinical sector associated costs.

## Conclusion

The current review covered a wide range of studies exploring the implications of the BDNF geneVal66Met polymorphism in schizophrenia, published in the last 5 years. The Met/Met genotype was found to be associated with higher PANSS positive scores, the PANSS global scores in these cases also reflecting greater severity of illness. It was also associated with a history of suicide attempts. Consistent with previous studies, Met/Met homozygous individuals present with worse cognitive function and lower levels of serum BDNF.

In the Val/Val genotype carriers, increased BDNF levels were correlated with weight gain under Risperidone treatment. However, due to the study’s small sample size depicting this, generalizations cannot be made. Future studies may take on the challenge of delineating genetic markers that could allow prediction for treatment response in schizophrenia.

## Data availability statement

The original contributions presented in the study are included in the article/supplementary material, further inquiries can be directed to the corresponding author.

## Author contributions

All authors listed have made a substantial, direct, and intellectual contribution to the work and approved it for publication.
